# Skip metastases to lateral cervical lymph nodes in differentiated thyroid cancer: a systematic review

**DOI:** 10.1186/s12893-018-0435-y

**Published:** 2019-04-24

**Authors:** Andrea Attard, Nunzia Cinzia Paladino, Attilio Ignazio Lo Monte, Nicola Falco, Giuseppina Melfa, Giulia Rotolo, Stefano Rizzuto, Eliana Gulotta, Giuseppe Salamone, Sebastiano Bonventre, Gregorio Scerrino, Gianfranco Cocorullo

**Affiliations:** 1 Policlinico “P. Giaccone”, Department of general emergency and transplant surgery, Unit of general and emergency surgery, Palermo, Italy; 2 Unit of General, Endocrine and Metabolic Surgery, CHU AP-HM Hôpital de la Conception (Marseille), 147 Boulevard Baille, 13385 Marseille cedex, France

**Keywords:** Papillary thyroid carcinoma, Lymph node dissection, Skip metastasis

## Abstract

Papillary thyroid carcinoma is a slow-growing cancer with a generally good prognosis that sometimes have an aggressive behaviour. Metastases to neck lymph nodes is the first step of the diffusion. The central neck compartment is involved most commonly. The ipsilateral lateral neck compartments are usually involved afterwards, and the involvement of the contralateral one is considered a quite rare occurrence. In more rare cases, metastases to lateral neck compartment without central lymph node metastasis (so called “skip metastases”) could be observed. Aim of this literature review study is to analyse the average incidence, pattern and risk factors of this occurrence.

This study was performed according to **PRISMA** criteria. A final selection of 13 articles published in English language from 1997 to 2017 was performed. Any research article, review or meta-analysis was taken into consideration. Research was expanded considering the related references of articles.

The incidence of skip metastases ranged from 1.6 to 21.8%. Risk factors such as age > 45 years, size < 5 mm and tumor located in the upper pole or isthmus of thyroid gland were found.

Due to the frequency of skip metastases in thyroid cancer, a careful preoperative examination of lateral lymph nodes should be necessary.

## Introduction

Papillary thyroid carcinoma (PTC) is slow-growing cancer and has generally a good prognosis for the majority of patients with a 5 years survival of 97–99% according to literature data [1–3].

Despite this excellent prognosis, some PTC and sometimes papillary thyroid microcarcinoma (PTMC), have an aggressive medical behaviour with an increase in mortality in some patients, as a consequence of molecular biology changes and other unclear causes [4–7].

Regional lymph node metastases are commonly observed in PTC and the risk of local regional recurrence occurs in 30–80% of cases [4, 8]. The central neck region is involved most commonly, increasing the overall morbidity and mortality as a potential consequence of increased risk of local recurrence [2, 5–7, 9, 10]. In spite of these observations, guidelines available up till now (2015 ATA guidelines) are cautious in extending indications for prophylactic central neck dissection (recommendation 36) and, on the other hand, restrict indication for lateral neck dissection only in the presence of biopsy-proven metastatic lateral compartment involvement (recommendation 37) [11].

According to the majority of papers available in literature, routine prophylactic node dissection is not indicated for all patients with PTC, in particular in small size tumors (T1 and T2) so pathological lymph nodes should be systematically identified preoperatively to avoid reoperation associated with a higher complication rate [2, 3].

The first anatomical site involved is the central compartment followed by the ipsilateral lateral compartment. Controlateral lateral compartment seems to be rarely involved [7, 12, 13].

The surgical treatment depends on the characteristics and evolution of PTC [5].

The risk factors of cervical metastases would appear to be the following: young age (< 45 years), multifocality, tumor size (> 0,5 cm) and site [6].

Zhang et al. [14] showed that the rate of lateral neck in the PTMC are located more frequently in upper pole than in middle portion of thyroid gland; in their experience PTMC located in upper pole of thyroid gland was associated with a reduced incidence of central lymph nodes metastases.

Xiang et al. [6] find 57.5% of central metastases in the PTMC located in the middle third of the thyroid gland.

Other authors emphasize a correlation with PTC located in the upper pole and development of lateral neck metastases [6, 7, 14, 15].

However, controversies remain regarding the correlation between tumor location and neck metastasis in PTMC [6].

Total Thyroidectomy with central neck dissection (CND) is recommended for patients with PTC according current guidelines but [6, 16] controversies still exists about the extension of cervical lymphadenectomy [6].

Despite the behaviour, some patients develop lateral lymph node metastasis without central lymph node metastasis. A lateral metastasis in the absence of a central one is defined skip metastasis.

According to literature, skip metastases occur with the dissemination of PTC cells through the lymphatic system gradually [5, 13].

Its incidence is variable according different studies.

In Jianyong Lei et al. experience, the incidence of skip metastases ranged from 6.8–37.5%.

According to other authors, this incidence varies between 11.1 and 37.5% [13].

In literature, many articles describe cases of skip metastasis but their behaviour unpredictable and small patients sample reported make their characterization difficult.

The present study revised the literature concerning skip metastases, with the aim of analysing average incidence, their pattern and risk factors.

## Materials and methods

This study was performed according to **PRISMA** criteria [17]. According to these guidelines, the selection of papers included has been carried out as follows.

### Literature search strategy

A systematic and comprehensive review of full texts was realized with literature research on PubMed. It included articles published in English language from 1997 to 2017. Medical Subject Heading (MESH) used for internet research were: “thyroid skip metastases”, “recurrence thyroid cancer”, “papillary thyroid carcinoma metastases”, “lymphadenectomy” with the Boolean operators AND or OR. In a first step of recruitment, all titles and abstracts of the identified studies for inclusion were evaluated. In a second phase a screening of full-text concerned the qualitative assessment of studies.

### Selection criteria

Clinical trials, systematic review and meta-analysis articles were included. Case reports were considered only in the presence of substantial theoretical knowledge. According to a collective decision of all Authors of this manuscript, we included in the systematic review all articles that reported incidence and described behaviour and risk factors of skip metastases. Our research was expanded considering the related references of articles. Articles concerning poorly differentiated or undifferentiated carcinomas were excluded. Articles concerning pediatric patients, concomitant diseases such as hyperparathyroidism, tumours arising from organs different from thyroid gland were also excluded.

### Study selection

**Fig. 1:**
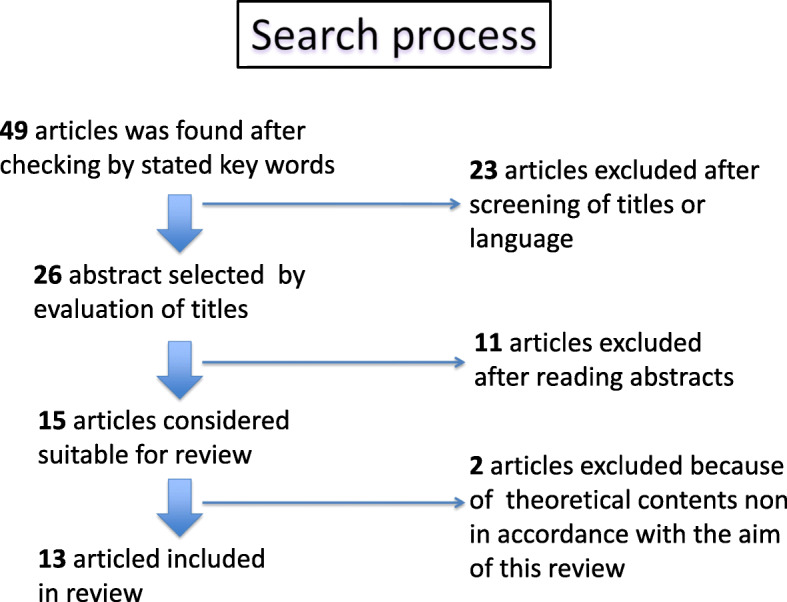
Screening of search results

At first, a total of 49 articles were considered in compliance with search strategy. The search process, that led to the final 13 selected articles was carried out as in Fig. 1.

## Literature analysis

### Incidence

The incidence of skip metastases varies in a wide range. Some studies analyse only its occurrence: Lee et al. [9] between 55 PTC analysed found 3 skip metastases; there are no other data on these 3 cases; Roh et al. [4] analysed 22 patients who underwent surgery for lateral nodal recurrence of PTC and found three skip metastases (14%). In another study the same Author [2], among 52 patients that underwent in all cases total thyroidectomy, CND and lateral neck dissection, found five skip metastases. (9.6%). According to Lei et al. [6], its incidence is 8.7%.

Xiang et al. [6] reports 11 skip metastases in 949 PTMC included (1.6%). A recent article of Lei et al. [7] reported an incidence of 39 skip metastases in 450 patients analysed (8.7%). To the best of our knowledge, it is the biggest case studies of skip metastases in literature. Zhan et al. [18] found 29 skip metastases among 272 patients enrolled (10.6%). Nie et al. [5] in their series of 261 patients with PTC found 30 (14.8%) skip metastases. Park et al. [15] found 32 skip metastases in 147 patients (21.8%). Chung et al. [18] included 245 patients with PTC. Among these patients, in 39 undergoing CND *plus* lateral neck dissection (LND), the authors found 12 with lateral metastases and 3 skip metastases (7.7%).

The biggest series published in the literature are reported in the following three articles that we want to highlight: Wada et al. [19] in 231 patients included, all treated with total thyropoidectomy (TT), CND and ipsilateral lateral dissection, found 17 skip metastases; Xiao et al. [20] enrolled 121 patients that underwent TT with CND and LND and they found nine skip metastases. In this study, a rate of incidence of 14.1% (9/64 lateral metastases) was reported.

Concerning the incidence, there is a wide variability from study to study.

Park et al. [15] report an average of 21.8% and finally Nie et al. [5] find an incidence of skip metastases of 14.8%.

In summary, in all studies consulted the incidence of skip metastases ranges from 1.6 to 21.8% (Table 1).

**Table 1:** Studies published

Authors	Years	N° Patients	Type of Surgery TT+		Skip metastases	Tumor site		Tumor size		Capsular invasion
			CND	CND + LND	N°	Upper pole	Middle portion	< 10	> 10	
Ducci M	1997	36	36	50 ispilateral 14 controlateral	11.1%	NE	NE	NE	NE	NE
Machens A	2004	215	215	95.3% ipsilateral 73.5% controlateral	13 (19.7%)	NS	NS	NS	NS	NS
Roh JL	2007	22	22	NS	3 (13,7%)	NE	NE	NE	NE	NE
Roh JL	2008	52	52	57	5	NS	NS	NS	NS	NS
Wada N	2008	231	231	231 ipsilateral	17	NS	NS	NS	NS	NS
Chung IS	2009	245	245	39 (12 metastases)	3					
Xiao GZ	2010	121	121	121	9	NS	NS	NS	NS	NS
Park JH	2012	147	147	147	32 (21.8%)	NS	NS	32	NS	NS
Xiang D	2015	949	949	44	11	8	3	NS	NS	NS
Lei J	2017	450	450	450	39 (8.7%)	37 (94.9%)	2 (5.1%)	71.8%	NS	84.6%
Zhan X	2017	272	TT	NS	29 (10.7%)	95%	NS	95%	NS	NS
Xlin N	2017	203	203	203	30 (14.8%)	19				
Lee B	2017	55	NS	55	3	NS	NS	NS	NS	NS

*TT* Total Thyroidectomy, *CND* central neck dissection, *LND* Lateral lymphoadenectomy, *NE* non evaluated, *NS* non specified

### Risk factors

From the analysis of literature, it would seem that site, size and capsular invasion of primary cancer could affect development of skip metastases. In support of this hypothesis, the article of Lei (6) showed that primitive PTC commonly had capsular invasion (84.6% vs 52.8%, *P* > 0.001), it was PTMC (71.8% vs 16.3%, *P* > 0.001), as confirmed in other articles [19] and most frequently was located in the upper thyroid portion except in two cases that were located in the middle portion. When all three risk factors (primary tumor located in the upper thyroid portion, tumor size < 10 mm and capsular invasion) were associated, the authors demonstrate the predictability of skip metastasis with the specificity of 0.98 and the Area Under Curve (AUC) was 0.7.

The importance of site of primary cancer is also emphasized by Xiang [6]. In this study, CND was realized for all patients. For bilateral and multifocal tumors or cancer located in the isthmus, ipsilateral CND was performed (236 patients). Central neck metastases were found in 299 patients (31.5%) and lateral neck metastases in 44 (4.6%). These 44 patients had lymphadenectomy from II to IV level. The skip metastases occurs only in tumors located in the upper poles (8 patients) and in middle portion of thyroid gland (3 patients).

These results confirm that the tumor site is an important risk factor in the development of skip metastases. In this analysis, owing to small number of skip metastases found, no statistical conclusions were made. Chung [18] did not find any risk factor associated to skip metastases, probably because of the small number of cases of CND *or* LND (*n* = 3) and CND + LND (*n* = 9).

Zhan and coll [21] also underline the importance of association between tumor size (< 10 mm), tumor site (upper pole of thyroid gland) and age of patients (> 45 years old) to develop skip metastases.In their experience, 95% of patients with skip metastases had a PTC located in the upper pole.

In the article of Nie [5] the inverse relation between tumor size and incidence of skip metastases was emphasized: in fact, the Author found it more frequently in PTMC especially when tumor size was ≤ 5 mm.

Park and coll. [15] report that skip metastases were less common (12.5% vs 30.4%) with multifocal tumors. Moreover, there were no differences in terms of age, sex, tumor bilaterality, tumor extension in the group with and without skip metastases. On the contrary, a significant association between skip metastases, primary tumor location and size was showed. Tis could be explained by the possible correlation between skip metastases and tumor located in the upper pole for possibly migration via lymphatic drainage accompanying the superior thyroid artery. In this study the primary tumor was < 1 cm, and presented single focus.

As regards as histology of primary tumor, from 215 patients included by Machens et al. [13] 66 were papillary, 8 follicular and 141 were medullary thyroid cancer. The Authors found 13 skip metastases (19.7%) in 66 papillary thyroid cancers and 30 (21.3%) in 141 medullary thyroid cancer.

Finally, capsular invasion and age > 45 years are possible risk factors underlined in some studies [18, 21].

### Pattern and localization of metastases

Skip metastases showed most frequently Level II metastases and less usually Level III, IV and V metastases. The PTC skip metastases showed a very high number of single-level metastases in the lateral compartment more rarely triple-and quadruple-level metastases [6, 7].

According to Park [15] the group with skip metastases had an inferior number of metastases of lateral nodes (3.3 ± 2.5 vs. 5.8 ± 3.5; *p* < 0,001) probably because these were found more frequently at a single level (53.1% vs. 20%; *p* < 0.001). In the group with skip metastases, these were found more frequently at levels III or II, a single cervical level and only a single node were involved. In support of the pathophysiologic hypothesis concerning the role of lymphatic drainage running close the superior thyroid artery, the levels III and II were most frequently involved in patients with skip metastases, so primary tumor located in upper pole tends to metastasize to upper levels rather than to the others levels.

## Discussion

The causes leading a metastasis to skip the central compartment are not completely clarified yet. At present, only risks factors could be invoked and few pathogenetic mechanism would be assumed.

Zhang et al. [14] underlined the importance of age  > 45 years (95%), presence of microcarcinoma (95%), tumor site (cancers located in the upper pole of the thyroid gland showed incidence of 95%).

To predict a possible existence of skip metastases and to know their natural history could help the surgeon in establishing surgical strategy, performing total thyroidectomy with adequate lymphectomy especially avoiding reoperations for recurrence, with an increase in morbidity and a worsening in prognosis.

Tumor locations, age, sex and tumor size can be considered strong risk factors and they can be useful in preoperative stratification of patients.

In the most of articles concerning skip metastases, the first tumor is PTMC.

In a recent analysis is proven that tumor size > 0.5 is correlated with both central and lateral metastases. In this analysis tumor size > 0.5 cm is an important risk factor [7].

Other authors [5] underline the correlation with tumor size less than 0.5 cm in diameter and skip metastases. In this analysis the authors show that the primary tumor location in the context of the upper pole of thyroid gland was significantly associated with lateral lymph node metastases (*p* = 0.000, OR = 10.471, 95%). In detail, in this analysis, a tumor located in the upper pole was more aggressive and it was correlated to development of skip metastases. This correlation can be explained by the lymphatic drainage system. Thyroid gland has rich lymphatic drainage [9]. Most usually affected lymph node levels in differentiated thyroid carcinoma are those of the anterior compartment group (VI) and the internal jugular chain (II-III-IV); less often those of the posterior triangle group (V); submental (Ia) and submandibular (Ib) nodes are only exceptionally involved (Fig. 1). An impressive work on cadaveric dissection was performed and previously published in Japanese literature by Sato et al. in 1994 [22] and subsequently published in English for the first time by Likhterov et al. in 2017 [23]. In this publication, the lymphatic system draining the thyroid gland was closely evaluated to explain the patterns of metastatic spread in patients with PTC. The anatomy of lymphatic channels coming from the gland explains the different ways of DTC metastasization to cervical lymph nodes. Although the central compartment lymph nodes seem to be the first step of lymphatic drainage before tumour cell transit to the lateral compartment, lymphatic drainage through superior lymphatic channels is directed from the central neck directly to levels II and III of the lateral neck; this explains the possibility of skip methastases occurrence.

Some authors [24–26] have reported that the lobes of thyroid gland have its own internal lymphatic system and no communication with controlateral regional lymph nodes. Although it is generally accepted that lymphatic drainage occurs from the thyroid primarily to the central lymphatic compartment and secondarily to the lateral compartment nodes, there are not many data about skip metastases and they are unpredictable.

Cervical metastasis occurs approximately in 35–80% of cases of PTC. They increase the risk of locoregional tumor recurrence and reoperation [9].

We would underline that most of selected studies have some limitations ad biases: as showed in Table 1, most papers did not report data about tumor size, site and capsular invasion.

As regards as treatment guidelines, complication commonly experienced from surgeons [27–35] that are more frequent after more extended neck procedures compared to standard thyroidectomy could influence in its turn the choice of performing a neck dissection in the absence of proven localization at central or lateral lymph nodes. Finally, it should be taken into consideration that in high-risk patients lateral lymph node involvement seems to be more frequent [36–38].

## Conclusion

In summary, according to this literature review, we can define “the high risk patient” by the following features: papillary or medullary PTMC located in upper pole or isthmus, unifocal. Due to frequency of skip metastases, that are rarely but not unusually found, a meticulous preoperative examination of all lateral lymph node compartments, especially in a typical “high risk patient”, should be necessary. In these patients the lymph nodes suspected for metastatic involvement should be identified at ultrasound exam, a biopsy should be also performed Moreover, a dosage of thyroglobulin in the eluate could confirm the diagnosis. Owing to the typical involvement of a single level a selective lateral neck dissection of the involved compartment seems to be an acceptable choice in most cases. The role of systematic lymphadenectomy is controversial considering the low aggressiveness of the PTC. However, it is necessary to distinguish patients at high risk to develop recurrences.

Total Thyroidectomy with extended lymphadenectomy may be indicated in high-risk patients, in whom lymph nodal recurrence is more frequent.

## Abbreviations

AUC: Area under curve
CND: Central neck dissection
LND: Lateral neck dissection
MESH: Medical subject heading
PTC: Papillary thyroid carcinoma
PTMC: Papillary thyroid microcarcinoma
TT: Total thyroidectomy
